# Comparative analysis of energy system contributions and physiological profiles in snowboard slopestyle and snowboard cross

**DOI:** 10.7717/peerj.20948

**Published:** 2026-03-19

**Authors:** Dae-hee Kim, Geonwoo Yang, Seung-Bo Park

**Affiliations:** 1 Graduate School of Sports Medicine, CHA University, Seongnam-si, Gyeonggi-do, Republic of South Korea; 2 Medical Center Research Institute, CHA Bundang Medical Center, CHA University, Seongnam-si, Gyeonggi-do, Republic of South Korea

**Keywords:** Snowboarding, Snowboard cross, Slopestyle, Energy system contributions, Physiological profiles

## Abstract

**Background:**

Slopestyle and snowboard cross are distinct snowboarding disciplines, each characterized by specific technical elements and differing competition dynamics. Examining the corresponding contributions of energy systems and physiological responses in these disciplines will yield essential foundational data to inform both physical conditioning programs and competitive strategies. This study compares the relative contributions of energy metabolism systems and physiological indices between the two disciplines.

**Methods:**

A crossover design was applied to eight elite male athletes registered with the Korea Ski & Snowboard Association; the athletes performed one simulated competition run each for slopestyle and snowboard cross. Oxygen consumption (VO_2_), heart rate (HR), and blood lactate concentration (La^−^) were measured during the runs, and the contribution of each energy system was calculated based on VO_2_ during exercise (oxidative), peak lactate concentration (glycolytic), and excess post-exercise oxygen consumption (phosphagen).

**Results:**

Compared with slopestyle, snowboard cross showed a significantly higher contribution from the glycolytic system (18.6% *vs.* 12.9%), higher peak lactate concentration (4.17 *vs.* 3.02 mmol L^−1^), and higher peak HR (168 *vs.* 158 bpm; *p* < 0.05). The mean run times were 66 s for snowboard cross and 75 s for slopestyle. Conversely, the contributions of the oxidative and phosphagen systems in slopestyle and snowboard cross were around 36.2–27.9% and 50.9–53.6%, respectively, showing no significant difference between the two disciplines; the total energy consumption (W_Total_) was also similar between the two disciplines (*p* > 0.05).

**Conclusion:**

Due to repeated high-intensity movements, snowboard cross is associated with a higher physiological load, as reflected by greater reliance on anaerobic glycolysis and higher lactate concentration and peak heart rate, whereas slopestyle tends to emphasize the use of oxidative metabolism owing to the recovery intervals between obstacles. However, this oxidative contribution did not differ significantly between disciplines. These results highlight the importance of establishing customized fitness training and recovery strategies based on the competition characteristics of each discipline.

## Introduction

Snowboarding is an extreme winter sport, with continual technical and competitive development; snowboard cross (SBX) was included in the Olympics in 2006 and slopestyle (SS) in 2014 (Platzer et al., 2009). Snowboarding competitions are broadly divided into the freestyle, alpine, and SBX disciplines. Of these, SS and SBX show clear differences in the course composition and method of execution. SS is an artistic discipline in which athletes perform a series of highly difficult technical maneuvers using various terrain features (rails, boxes, kickers, *etc.*), and the winner is decided based on judges’ scores. However, SBX is a speed-based racing discipline in which multiple athletes start simultaneously; the first to complete a course, which includes a series of obstacles such as banks, jumps, and rollers, is declared the winner (Federolf et al., 2008; Platzer et al., 2009). Athletes participating in SS and SBX navigate their courses at high velocities, encountering obstacles. SS places significant emphasis on technical execution and creative expression, whereas SBX prioritizes velocity and competitive positioning (Wang, Zhong & Wang, 2023).

As performance difficulty increases, snowboarding requires precise upper and lower body control and rapid muscular force production, leading to substantial physiological demands (Vernillo, Pisoni & Thiébat, 2016). In both SS and SBX, repeated high-intensity intermittent actions place considerable demands on both anaerobic capacity and aerobic endurance (KC JU, 1997; Platzer et al., 2009; Thomas, Hynes & Ingledew, 2002; Vernillo, Pisoni & Thiébat, 2018; Zebrowska et al., 2012). In SS, explosive technical maneuvers are interspersed with low-intensity gliding phases. In contrast, SBX consists of repeated high-intensity intermittent actions to generate and maintain speed throughout the course (Federolf et al., 2008; Platzer et al., 2009). SS involves rotational and directional movements, whereas SBX predominantly consists of vertical unweighting, absorption, and pumping actions (Platzer et al., 2009). These high-intensity, rapid movements primarily rely on energy from the phosphagen and glycolytic systems, while during relatively low-intensity intervals, such as gliding after landing from a jump, the contribution of the body’s oxidative system appears to increase (Franchini, 2023; McPhail et al., 2024; Platzer et al., 2009). Although most elite athletes specialize in a single discipline, clarifying discipline-specific physiological demands is important. This information can provide scientific evidence to support coaches, strength and conditioning specialists, and sport scientists in designing more targeted training strategies, and can also help elite athletes identify performance limitations and optimize their preparation according to the specific demands of each discipline (Wang, Zhong & Wang, 2023). In high-intensity intermittent sports, the contribution of the body’s oxidative metabolism system is generally greatest over the whole duration of the competition, while anaerobic pathways tend to be used especially at decisive moments (Gastin, 2001). For example, in Olympic boxing, around 60–90% of the total energy is supplied *via* oxidative pathways, while the contributions of the phosphagen and glycolytic systems are reported to be around 10–30% and 20% or less, respectively (Franchini, 2023). In other words, these high-intensity sports mostly depend on the oxidative system for energy supply; however, the explosive movements that influence the outcomes are supported by anaerobic pathways, such as the phosphagen and glycolytic systems (Beneke et al., 2004).

In SS and SBX competitions as well, energy supply patterns differ with rapid fluctuations in exercise intensity (Platzer et al., 2009). When exercise intensity peaks, the anaerobic system is predominantly recruited for immediate adenosine triphosphate (ATP) resynthesis (Seifert, Kröll & Müller, 2009). While the phosphagen system provides the highest energy output during maximal exertion, its limited intramuscular phosphocreatine (PCr) capacity necessitates additional ATP production *via* glycolysis, which typically supports high-intensity activity lasting 10–90 s (Gaitanos et al., 1993; Hargreaves & Spriet, 2020; Hauser, Adam & Schulz, 2014; Hirvonen et al., 1987; Quittmann et al., 2020; Sauer & Schlattner, 2004). However, excessive lactate accumulation impairs muscle function and contributes to fatigue (Beneke et al., 2005; Brooks, 2018; Faude, Kindermann & Meyer, 2009; Quittmann et al., 2020). Thus, although anaerobic pathways are crucial for explosive actions, oxidative metabolism during short recovery intervals is equally essential to sustain performance throughout the run.

Training methods based on analysis of energy system usage, which depends on competition characteristics, have been studied and introduced for some winter sports. However, studies investigating the physiological characteristics of snowboard disciplines are rare, especially those comparing metabolic demands between disciplines with different characteristics, such as SS and SBX. To date, no study has directly compared the energy system contributions of these two disciplines within the same athletes. This absence of athlete-matched comparisons represents a significant gap in understanding discipline-specific metabolic demands (KC JU, 1997; Losnegard, 2019). To our knowledge, no previous study has directly compared these two Olympic disciplines in elite athletes—largely owing to the practical difficulty of recruiting participants who can perform both events. Thus, the present study represents a novel attempt to address this gap by highlighting the discipline-specific physiological and metabolic profiles of SS and SBX.

This study investigates differences in the physiological demands between SS and SBX by quantitatively calculating the relative contributions of three energy metabolism systems (phosphagen, glycolytic, and oxidative) during competition, in light of the differing characteristics of each discipline. To this end, we measured physiological indices, such as the blood lactate concentration (La^−^), oxygen consumption (VO_2_), and heart rate (HR) immediately after competition. We hypothesized that the contribution of an anaerobic energy system would be higher in SBX than in SS. This hypothesis was based on the requirement for repeated high-intensity efforts to generate speed in SBX, which suggests greater reliance on anaerobic pathways (Earp, Hatfield & Downing, 2021; Platzer et al., 2009).

## Methods

### Participants

In this study, eight South Korean elite male snowboarders officially registered with the Korea Ski & Snowboard Association volunteered to participate. The sample size was determined *a priori* using G*Power 3.1.9.4, which indicated that *N* = 8 would be sufficient to detect a large within-subject effect (dz = 1.16) with *α* = 0.05 and power = 0.80 in a paired samples *t*-test. The participants had been active athletes for at least 3 years and performed regular training for at least 10–15 h per week. The participants’ characteristics are shown in Table 1.

All participants were elite snowboarders with competitive experience in both SS and SBX at the national level. Among them, four riders had recorded rankings in domestic SBX competitions, while the other four had achieved results in domestic SS competitions. According to the Participant Classification Framework (McKay et al., 2021), all riders correspond to Tier 3 (Highly Trained/National Level), with three also having *Fédération Internationale de Ski et de Snowboard* (FIS) competition experience internationally but without top rankings.

The participants did not take any medications during the test period, abstained from vigorous exercise and alcohol for 48 h and caffeine and analgesics for 6 h before each experiment, and obtained at least 6 h sleep the night before the experiment. All participants were thoroughly informed of the study purpose and procedures before the experiment and provided written consent. For participants who were minors, consent was provided by their parents or legal guardian. This study was approved by the Chung-Ang University (Seoul, South Korea) institutional review board (1041078-20250131-BR-023) and adhered to the principles of the Declaration of Helsinki.

### Study design

**Figure 1:**
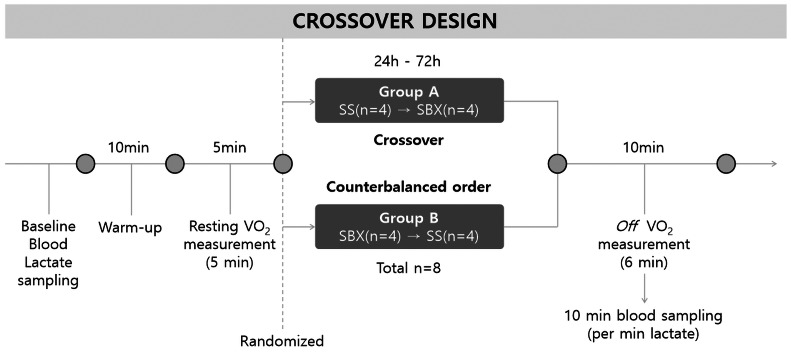
Schematic representation of the crossover study design. SS, slopestyle; SBX, snowboard cross; *Off* VO_2_, oxygen consumption after SS or SBX.

This study used a randomized, controlled, crossover design to compare the energy metabolism responses between the SS and SBX disciplines. To minimize potential performance bias, participants were not informed of the specific study hypotheses. To control for potential ordering effects, we employed a counterbalanced crossover design, randomly allocating participants to SS and SBX conditions (Fig. 1).

### Study procedure

This study consisted of two separate experiments (SS and SBX), each conducted with the same participants. To enhance the validity of the results, each course was completed five times, with intervals of at least 24 h and at most 3 days between each session. During this process, participants were also familiarized with the measurement procedures. All physiological data were collected during one official simulated run that was performed after sufficient familiarization trials.

To analyze resting lactate for each discipline, 20 µL of capillary blood was collected from the earlobe of each participant before warming up (mmol L^−^^1^; [La^−^]_(Rest)_). The collected blood samples were analyzed using an enzyme electrochemical method (Biosen C-line, EKF Diagnostics GmbH, Barleben, Germany) (Losnegard, 2019; Quittmann et al., 2020). After completing a warm-up, the participants rested for 5 min before being fitted with a portable respiratory gas analyzer (VO_2_ Master Analyzer; VO_2_ Master Health Inc., Vernon, BC, Canada) and Polar H10 HR monitor (Polar Electro Oy, Kempele, Finland) to measure VO_2_ and HR, respectively. VO_2_ (mL kg^−^^1^ min^−^^1^) was measured per breath using a VO_2_ Master. The respiratory gas analyzer was calibrated using flow calibration and manual verification, and the turbine volume converter was calibrated using a 3-L syringe (Hans Rudolph, Kansas City, MO, USA). Performance in SS was evaluated based on skill execution, whereas SBX was assessed through individual race performance. To avoid interference with the lactate accumulated in the blood, participants then rested in a seated position for passive recovery. VO_2_ was measured for 6 min after performing each discipline. Peak oxygen consumption (VO_2peak_) values were determined from oxygen consumption data collected post-course (VO_2post_). While the participants were recovering, capillary blood was collected from the earlobe to measure the peak lactate concentration ([La^−^]_max_) after course completion at 1-min intervals for 10 min (Quittmann et al., 2020). Differences in lactate concentration were calculated by subtracting resting lactate level from peak lactate level.

**Table 1:** Participants’ characteristics.

KSA participants (*n* = 8)	Age years	Height cm	Weight kg	BMI kg/m^2^	Heart rate rest
(mean ± SD)	28.62 ± 11.82	177 ± 4.781	77.75 ± 10.96	24.84 ± 3.54	80.9 ± 7.5

**Notes.**

KSA Korea Ski & Snowboard Association

We followed FIS regulations for the SS and SBX courses, with one rider on the course at a time. All participants completed one official simulated run per discipline, reflecting the format of an actual competition. The main experiment was conducted at a ski resort in Gangwon-do, South Korea. At the time, the air temperature was 4–6 °C, and the relative humidity was 60%. The snow was mechanically compacted and had a high moisture content owing to the above-zero temperatures, resulting in wet snow.

#### Warm-up procedures by discipline

Before the experiment, all participants completed five familiarization sessions for each discipline. Each session included course inspection and repeated training runs to ensure that participants were fully accustomed to the experimental procedures and course settings. Standardized rest was provided: 5 min before each run and at least 6 min of seated recovery after each run. If a participant fell or was disqualified, the trial was repeated after rest. During all sessions and official runs, athletes wore protective equipment, including knee pads, padded shorts, body armor, helmets, goggles and gloves.

All participants performed warm-up exercises for 10 min, appropriate for each discipline and the course conditions. In the SBX session, pump tracks and wave parks were used to activate rhythm, terrain adaptation, center-of-mass shifts, and pumping skills required for the course. This warm-up was designed to allow the participants to adapt to the high-intensity, intermittent, repetitive movements required for the course. In the SS session, small obstacles (miniature rails and kickers) around the main course were used to practice basic sliding and jump entry. These exercises focused on minimizing technical errors related to obstacle entry angles, speed perception, and landing stability.

#### SS experimental procedure

**Figure 2:**
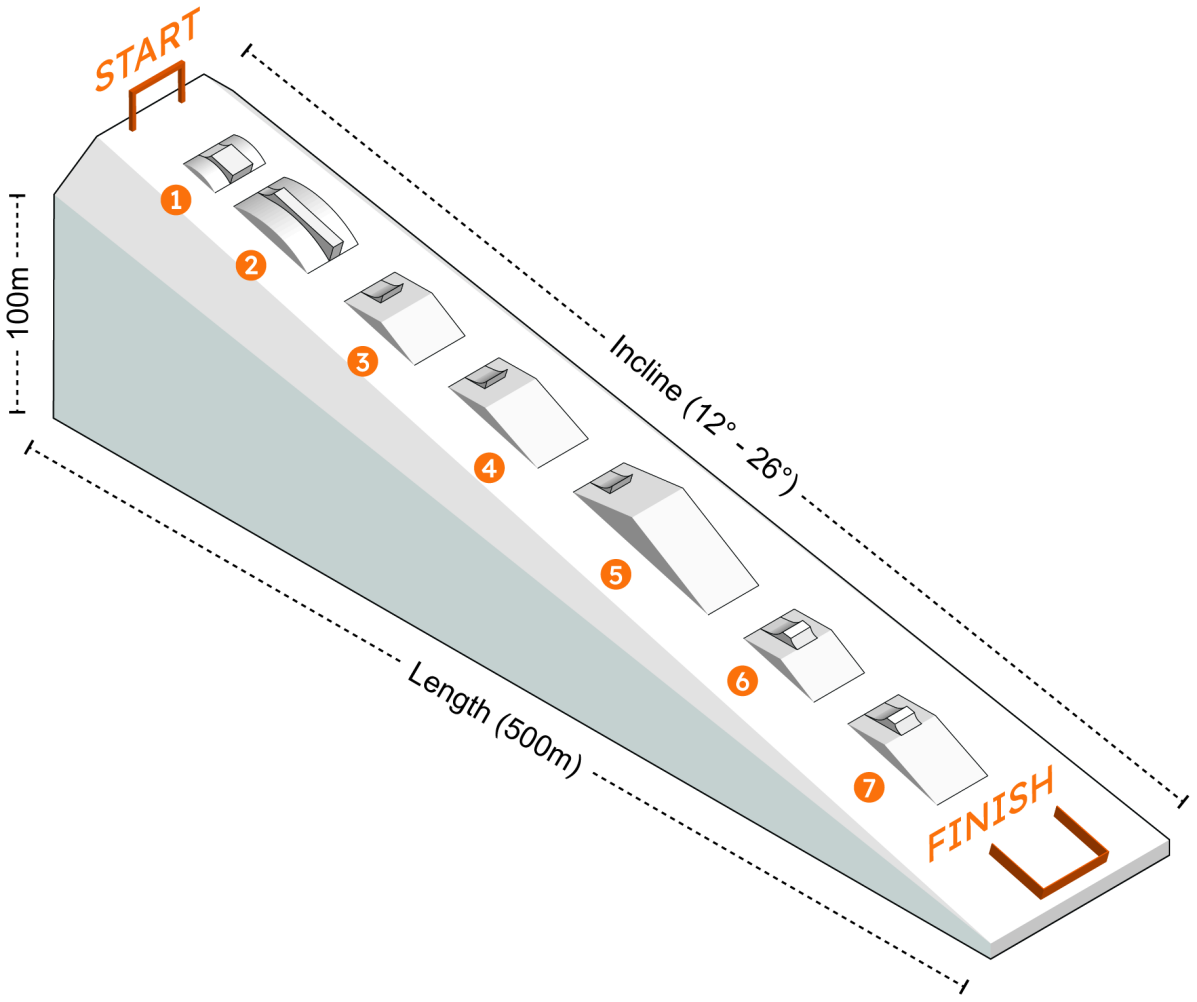
Diagram of the slopestyle course (length = 500 m; Incline = 12°–26°). Obstacles include: 1, wide box; 2, straight box; 3, kicker (4 m); 4, kicker (6 m); 5, main kicker (14 m); 6–7, table kickers (4 m and 6 m).

The SS course was designed in accordance with international specifications as a domestic FIS championship-level course, consisting of seven sections in total. It was composed of one straight box, one wide box, three main kickers, and two table kickers. The main kickers lengths were 6 m, 8 m, and 14 m, and were designed to enable various difficulties of jump and grind skills (Fig. 2). To minimize the potential influence of trick complexity on energy system contributions, all SS runs consisted only of basic straight-air jumps and non-rotational rail tricks, without any rotational elements (Merz et al., 2025).

#### SBX experimental procedure

**Figure 3:**
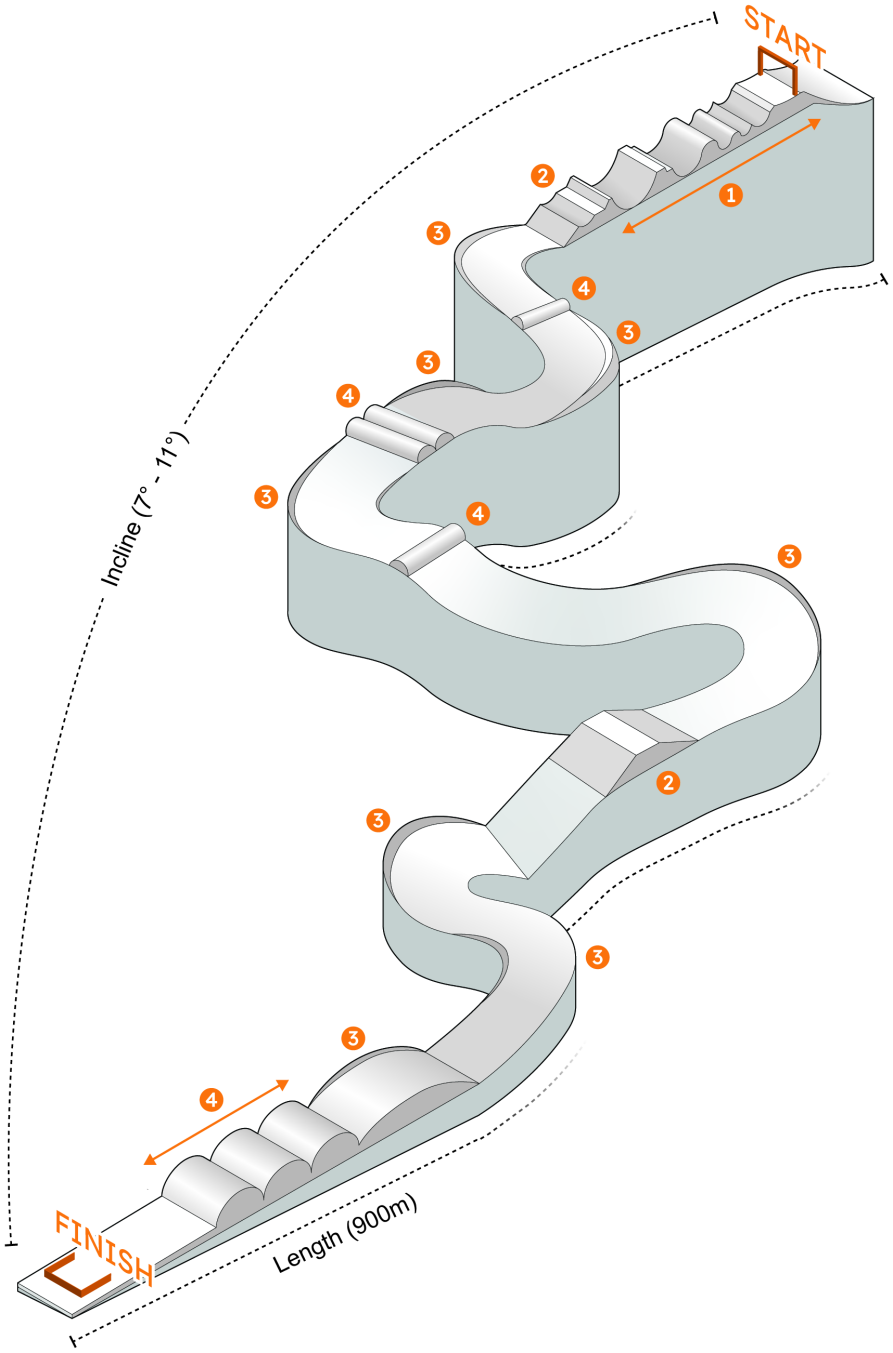
Diagram of the snowboard cross course (length = 900 m; Incline = 7°–11°). The course includes: 1, U-tank; 2, table kick; 3, banked turn; 4, wave section.

The SBX course was designed in accordance with international specifications as a domestic FIS championship-level course, consisting of 21 sections in total. The course included five U-shaped mounds, eight banks, seven waves, and two table kickers. This was designed to allow the athletes to experience high-speed sections and series of obstacles (Fig. 3). Runs were conducted by a single athlete at a time, and all competitions were based on one run. Each rider completed the course with maximal effort, as in actual competition, provided that they did not fall during the run.

### Physiological measurements

To assess the participants’ physiological responses, their HR, VO_2_, and blood lactate concentration were measured. HR was measured using a Polar H10 HR monitor. The peak HR and mean HR during the experiment were recorded. Oxygen consumption (VO_2_) was measured breath-by-breath using a portable gas analyzer (VO_2_ Master Analyzer, VO_2_ Master Inc., Canada). The validity and reliability of this device for field-based VO_2_ and ventilation measurements have been demonstrated in previous studies (Montoye, Vondrasek & Hancock, 2020; Seemann-Sinn et al., 2025), supporting its applicability in short, high-intensity outdoor exercise. Peak oxygen consumption (VO_2__peak_) and mean oxygen consumption (VO_2__mean_) were also measured. To analyze blood lactate concentration, 20 µL capillary blood samples were collected from the earlobe before the run (rest) and from 1 to 10 min post-run. The collected blood samples were analyzed using an enzyme electrochemical method (Biosen C-line, EKF Diagnostics GmbH).

### Calculation of energy system contributions

To measure the energy demand, the contributions of the various energy systems were assessed based on oxygen consumption before (VO_2Rest_), during (VO_2Snowboarding_), and after (VO_2post_ and La^−^) runs. The energy contributions of the phosphagen system, glycolytic system, and oxidative system (W_PCr_, W_Gly_, W_Oxi_) were calculated using the oxygen capacity measured during snowboarding (VO_2_), peak blood lactate concentration (Peak La^−^), and rapid component of excess post-exercise oxygen consumption (EPOC; excess VO_2_).

W_PCr_ was calculated based on the rapid component of the EPOC during the recovery phase after the run. W_PCr_ was determined by deducting the VO_2__Rest_ from the rapid component of VO_2__Post_, measured immediately after the run. The VO_2_ data from post-run sessions were suitable for a two-component model. W_PCr_ was calculated by integrating only the fast component of the two-component function (Archacki et al., 2024; Campos et al., 2012; De Campos Mello et al., 2009; Park et al., 2020).

\begin{eqnarray*}{\mathrm{W}}_{\mathrm{PCr}}{O}_{2} \left( L \right) = \frac{{A}_{1}}{60} \times ta{u}_{1}\times \frac{1}{1000} \end{eqnarray*}

\begin{eqnarray*}{\mathrm{W}}_{\mathrm{PCr}} \left( kJ \right) ={O}_{2} \left( L \right) \cdot 20.92 \end{eqnarray*}

*tau*_1_: Time constant (in seconds) corresponding to the fast component of post-exercise VO_2_ recovery.

*A*_1_: The amplitude of the fast component of EPOC.

W_Gly_ was calculated based on the blood lactate concentration measured immediately after the run. An increase of one mmol L^−^^1^ in lactate concentration was assumed to correspond to a consumption of three mL O_2_ per 1 kg body weight (Di Prampero & Ferretti, 1999; Duffield, Dawson & Goodman, 2005). The oxygen consumption calculated was converted from liters to energy units (kJ) based on the assumption that 1 L of O_2_ is equivalent to 20.92 kJ (Gastin, 2001). ΔLa^−^ was calculated as the difference in blood lactate concentration before and after snowboarding, where the post-run measurement was based on the highest measured concentration within 5 min of run completion (Beneke et al., 2004; Campos et al., 2012).

W_Gly_ = Δ[La^−^] (mmol L^−^^1^) ⋅*m*_*body*_(kg) ⋅[3 mL O_2_/(kg mmol^−^^1^)] ⋅(1/1000) (L/mL) ⋅20.92(kJ/L O_2_)

\begin{eqnarray*}Glycolytic~Energy \left( kJ \right) ={O}_{2}(L)\cdot 20.92 \end{eqnarray*}

*m*_*body*_ denotes the body weight.

W_Oxi_ was estimated by deducting the resting oxygen consumption (VO_2__Rest_) from the oxygen consumption measured during snowboarding (VO_2_). VO_2__Rest_ was defined as the mean value of the last 30 s of measurements taken during 5 min of rest (Beneke et al., 2004; Julio et al., 2017). The area below the oxygen consumption curve was calculated using the trapezoidal method (De Campos Mello et al., 2009).

\begin{eqnarray*}{W}_{Oxi}= \left( \sum VO_{2}^{segment} \right) \times 20.92 \end{eqnarray*}

\begin{eqnarray*}Oxidative~Energy \left( kJ \right) ={O}_{2}(L)\cdot 20.92 \end{eqnarray*}

$\sum V{O}_{2}^{segment}$ denotes the VO_2_ during exercise–resting VO_2_ in each exercise interval. The total energy of the system (W_Total_) was estimated as the sum (kJ) of the three energy systems analyzed (W_PCr_ +W_Gly_+ W_Oxi_) (Campos et al., 2012; De Campos Mello et al., 2009; Duffield, Dawson & Goodman, 2005; Park et al., 2020).

\begin{eqnarray*}{E}_{total}={E}_{oxi}+{E}_{gly}+{E}_{phos}. \end{eqnarray*}

### Statistical analysis

Statistical analysis was performed using a Python-based (v3.10) coding environment, and the main results were cross-checked in SPSS Statistics Version 27.0 (IBM Corp., Armonk, NY, USA) to ensure the validity and reproducibility of the analysis. All data are presented as mean ± standard deviation (*SD*). To compare physiological and performance variables between the SS and SBX conditions, paired samples *t*-tests were consistently applied to all continuous variables. This single analytical approach was chosen a priori owing to the well-established robustness of the *t*-test to moderate violations of the normality assumption, making it a powerful and appropriate method for the given sample size (*N* = 8). Statistical significance was set at *p* < 0.05.

## Results

### Participant characteristics

The physiological characteristics of the participants are summarized in Table 1. Baseline physiological indices such as mean age, height, body mass, and resting heart rate are presented as mean ± SD.

### Physiological parameters

VO_2Snowboarding_ and VO_2peak_ show a trend for being slightly higher in SBX, but the difference was not statistically significant (*p* > 0.05). In terms of the physiological response, peak La^−^ and peak HR were significantly higher in SBX than in SS (*p* < 0.05). With regard to the contributions of energy systems, the absolute and relative contributions of the W_Gly_ system (W_Gly_ kJ) were significantly higher in SBX (*p* < 0.05), while no significant differences were observed in W_PCr_ or W_Oxi_ (*p* > 0.05; Table 2).

**Table 2:** Physiological parameters and energy metabolism systems.

	SBX	SS	Significance	Effect size
Parameters	(mean ± SD)	(mean ± SD)	*p* value	*d* value
Trial Time	66.38 ± 6.25	75.25 ± 8.19	0.03^*^	−0.95
Peak HR	167.62 ± 14.63	157.75 ± 17.18	0.04^*^	0.89
W_PCr_ (kJ)	40.15 ± 12.77	33.68 ± 18.85	0.22	0.47
W_Gly_ (kJ)	13.92 ± 4.72	8.51 ± 3.29	0.02^*^	1.02
W_Oxi_ (kJ)	20.89 ± 4.09	23.94 ± 8.91	0.38	−0.33
W_Total_ (kJ)	74.96 ± 18.62	66.14 ± 27.81	0.30	0.40
W_Pcr_ (%)	53.6	50.9	0.32	−0.37
W_Gly_ (%)	18.6	12.9	0.20	−0.49
W_Oxi_ (%)	27.9	36.2	0.01^*^	1.10
Peak La^−^ (mmol L^−^^1^)	4.17 ± 1.10	3.02 ± 0.66	0.02^*^	0.97
ΔLa^−^ (mmol L^−^^1^)	2.90 ± 1.09	1.75 ± 0.69	0.03^*^	0.92
VO_2__baseline_ (mL⋅ kg^−^^1^⋅ min^−^^1^)	6.14 ± 0.45	5.46 ± 0.79	0.06	0.79
VO_2__Snowboarding_ (mL⋅ kg^−^^1^⋅ min^−^^1^)	17.73 ± 2.02	17.09 ± 4.06	0.63	0.18
VO_2__peak_ (mL⋅ kg^−^^1^⋅ min^−^^1^)	26.95 ± 4.27	25.21 ± 7.11	0.34	0.36

**Notes.**

* Data are presented as mean ± standard deviation (SD). *p* < 0.05 indicates statistical significance between conditions. Effect size (d) was calculated using Cohen’s d, with thresholds of 0.2 (small), 0.5 (medium), and 0.8 (large).

SS slopestyle SBX snowboard cross *Off* VO_2_ oxygen consumption after SS or SBX Peak HR peak heart rate W_PCr_ phosphagen contribution (kJ) W_Gly_ glycolytic contribution (kJ) W_Oxi_ oxidative contribution (kJ) W_Total_ total energy contribution (kJ) W_PCr_ phosphagen contribution (%) W_Gly_ glycolytic contribution (%) W_Oxi_ oxidative contribution (%) W_Total_ total energy contribution (kJ) Peak La^−^ peak blood lactate concentration (mmol L^−^^1^) ΔLa^−^ change in blood lactate concentration (mmol L^−^^1^) VO_2__baseline_ baseline oxygen consumption (mL kg^−^^1^ min^−^^1^) VO_2__Snowboarding_ oxygen consumption during snowboarding (mL kg^−^^1^ min^−^^1^) VO_2__peak_ peak oxygen consumption (mL kg^−^^1^ min^−^^1^)

### Blood lactate concentration

**Figure 4:**
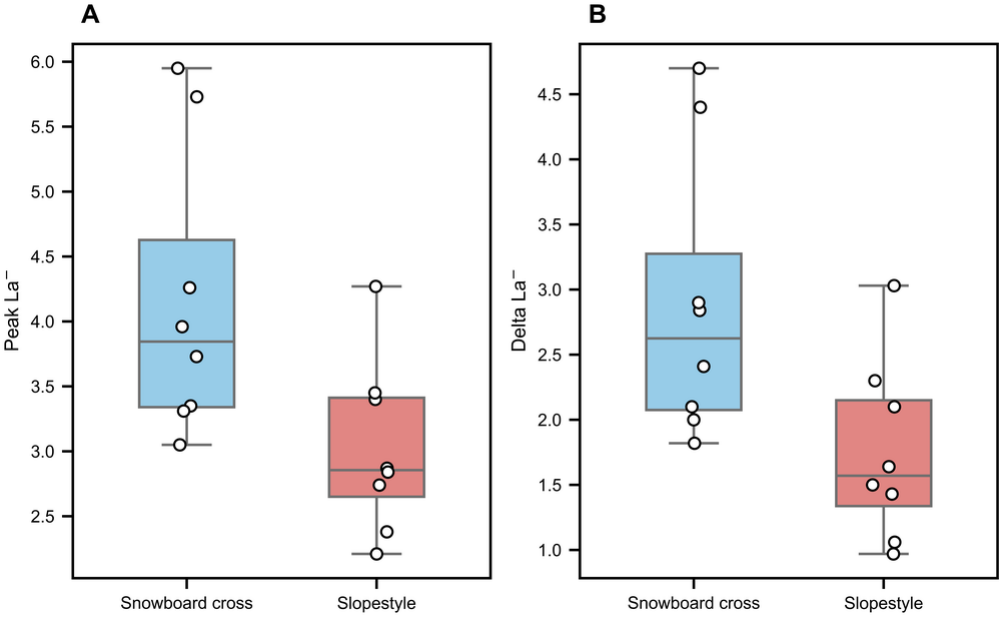
Blood lactate concentration during snowboard cross and slopestyle conditions: mean (±SD) values of the Peak La^−^ and Delta La^−^ for SS (red box) and SBX (blue box). (A) Peak La^−^; (B) Delta La^−^; each point represents the data of an individual participant, and the lines indicate the within-subject change between the two conditions for the same participant *p* < 0.05.

Peak La^−^ was significantly higher for SBX (4.17 ± 1.10 mmol L^−^^1^) than for SS (3.02 ± 0.66 mmol L^−^^1^; *p* < 0.05, *d* = 0.97) (Fig. 4A). In addition, ΔLa^−^ was also significantly higher for SBX (2.90 ± 1.09 mmol L^−^^1^) than for SS (1.75 ± 0.69 mmol L^−^^1^; *p* < 0.05, *d* = 0.92) (Fig. 4B).

### Heart rate

**Figure 5:**
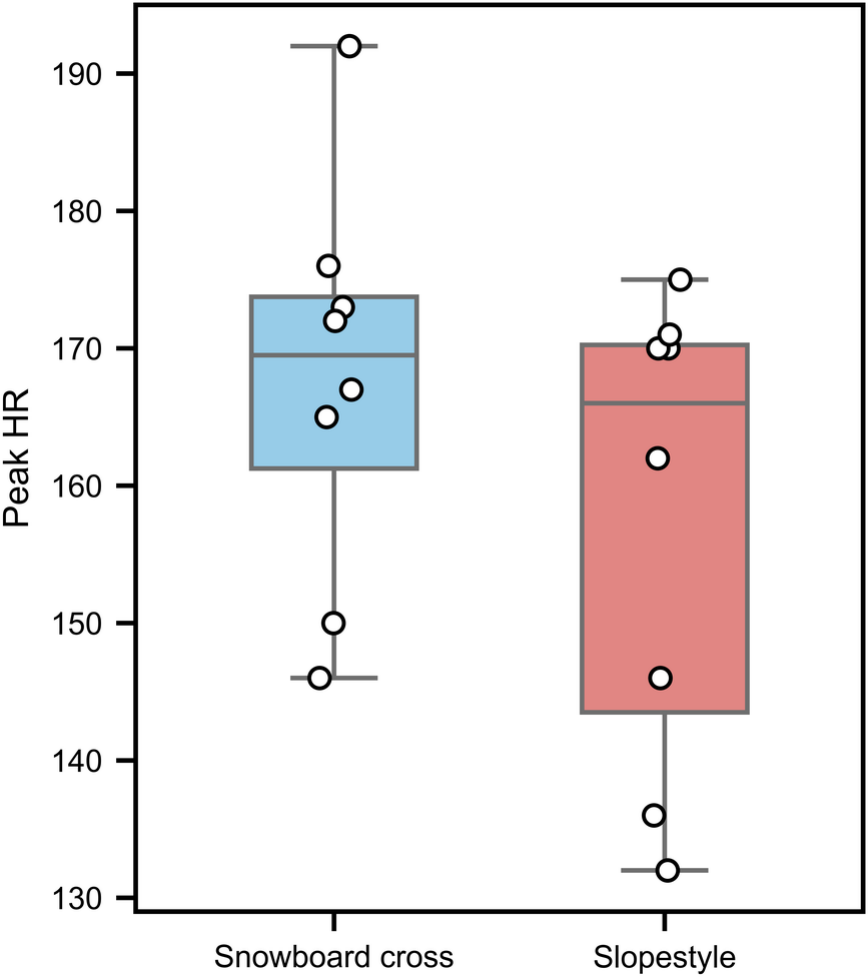
Peak heart rate during snowboard cross and slopestyle conditions: mean (±SD) values for slopestyle (red box) and snowboard cross (blue box). Each point represents the data of an individual participant, and the lines indicate the within-subject change between the two conditions *p* < 0.05.

As shown in Fig. 5, peak HR was significantly higher for SBX (167.62 ± 14.63 bpm) than for SS (157.75 ± 17.18 bpm; *p* < 0.05, *d* = 0.89).

### Trial time

**Figure 6:**
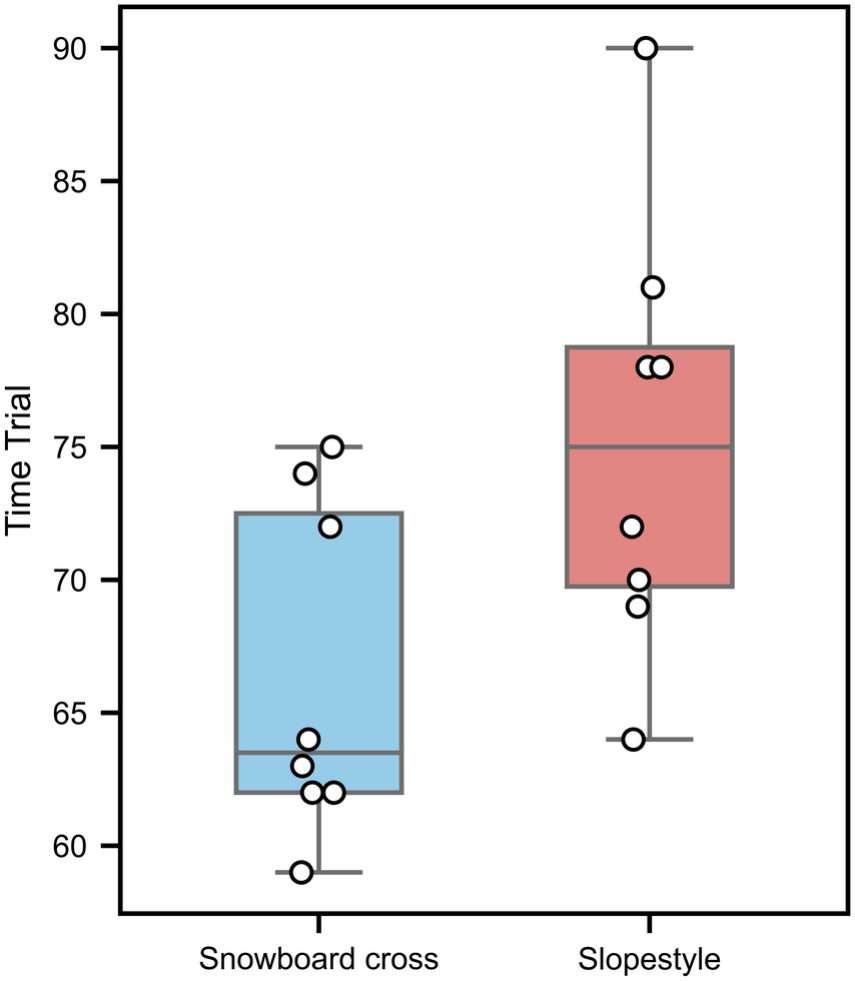
Trial times during snowboard cross and slopestyle conditions: mean (±SD) values for slopestyle (red box) and snowboard cross (blue box). Each point represents the data of an individual participant, and the lines indicate the within-subject change between the two conditions *p* < 0.05.

As shown in Fig. 6, the total run time was significantly less for SBX (66.38 ± 6.25 s) than for SS (75.25 ± 8.19 s; *p* < 0.05, *d* =  − 0.95).

### Energy metabolism systems

**Figure 7:**
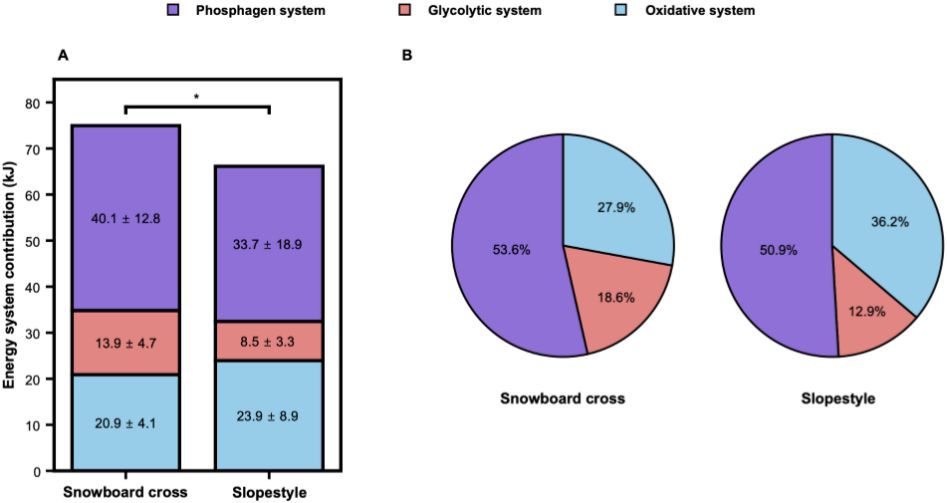
Absolute (A) and relative (B) energy system contributions during snowboard cross and slopestyle. Stacked bars (A) show absolute contributions (mean ± SD, kJ), and charts (B) show relative shares (%). Energy-system colors: blue, oxidative; red, glycolytic; purple, phosphagen. *p* < 0.05 indicates significant differences between groups.

**Figure 8:**
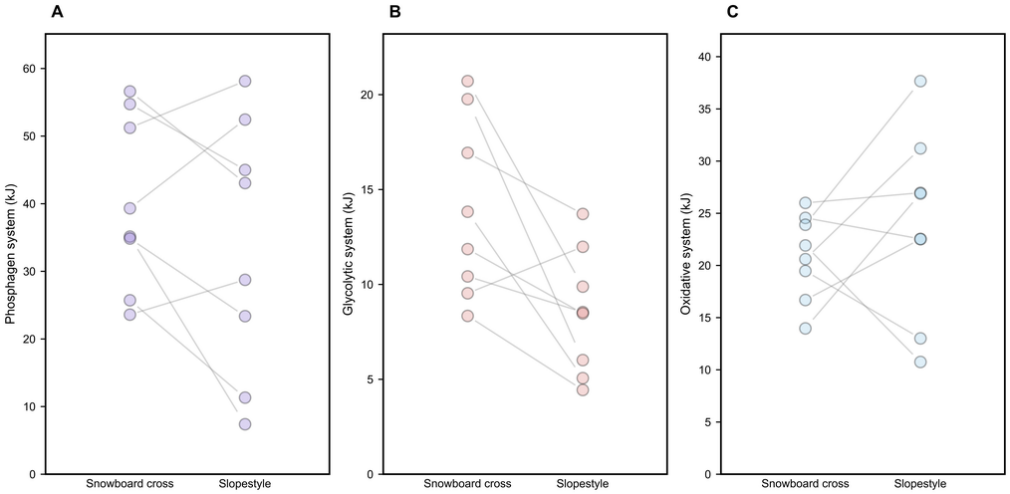
Absolute energy system contributions (kJ) in slopestyle and snowboard cross: represent mean ± SD values for each energy system. (A) phosphagen system; (B) glycolytic system; (C) oxidative system; each point represents the data of an individual participant. *p* < 0.05 indicates significant differences between the two conditions.

The absolute (kJ) and relative (%) energy system contributions are presented in Table 2 and Fig. 7. The individual data distributions are illustrated in Fig. 8.

The contribution of the W_PCr_ was higher for SBX (40.15 ± 12.77 kJ; 53.6%) than for SS (33.68 ± 18.85 kJ; 50.9%), but this difference did not reach statistical significance (*p* > 0.05, *d* = 0.47). The contribution of the W_Gly_ was higher for SBX (13.92 ± 4.72 kJ; 18.6%) than for SS (8.51 ± 3.29 kJ; 12.9%; *p* < 0.05, *d* = 1.02). The contribution of the W_Oxi_ was higher for SS (23.94 ± 8.91 kJ; 36.2%) than for SBX (20.89 ± 4.09 kJ; 27.9%), but this difference was not statistically significant (*p* > 0.05, *d* = −0.33). W_Total_ was not statistically significantly different between the two disciplines (*p* = 0.30, *d* = 0.40).

## Discussion

In this study, we compared physiological responses and energy system contributions in the snowboarding disciplines of SS and SBX. The main finding was that the contribution of the W_Gly_ system (*d* = 1.02, *r* = 0.77), Peak La^−^ (*d* = 0.97, *r* = 0.78), ΔLa^−^ (*d* = 0.92, *r* = 0.68), and peak HR (*d* = 0.89, *r* = 0.70) were significantly higher in SBX than in SS. The effect sizes (d and r) indicated large effects for W_Gly_, Peak La^−^, and peak HR (*r* > 0.70), and a medium-to-large effect for ΔLa^−^ (*r* = 0.68). Conversely, the contributions of the W_Oxi_ (*d* =  − 0.33, *r* = 0.30), W_PCr_ (*d* = 0.47, *r* = 0.48), and W_Total_ systems (*d* = 0.40, *r* = 0.37) were not statistically significantly different between the two disciplines. Mean run times were significantly longer for SS than for SBX. These results are thought to reflect differences in the physiological energy supply patterns required owing to the distinct athletic characteristics of each discipline (Platzer et al., 2009).

Due to the nature of SBX competitions, athletes accelerate rapidly as soon as the race starts and then have to pass through a series of obstacles, including banks, jumps, and rollers, at high speed. As such, the energy demand increases rapidly in a short time, and the W_Gly_ system is actively recruited for fast ATP supply (Bacharach & Von Duvillard, 1995; Hogg, 2003; Platzer et al., 2009). In other words, in SBX, the W_Gly_ system acts as the main energy pathway to sustain a chain of explosive movements; in SS, there are brief recovery periods, such as the relatively short gliding intervals between technical movements and preparatory intervals after landing. Owing to these athletic characteristics, continuous skill performance and precise postural control are more important in SS than momentary speed-based competition, and it appears that the W_Oxi_ energy system is used intermittently for rapid recovery before the next technical movement (Hogg, 2003; Platzer et al., 2009; Vernillo, Pisoni & Thiébat, 2016; Vernillo, Pisoni & Thiébat, 2018).

The difference in contributions of the W_Oxi_ system was not statistically significant in our study (*p* > 0.05, *d* = −0.33), while this finding does not allow for firm conclusions, one possible interpretation is that the increased oxygen consumption during competition indicates that aerobic oxygen supply was not meaningfully different between the two disciplines. The contribution of the W_PCr_ system also showed no significant difference between SS and SBX (*p* > 0.05, *d* = 0.402). This finding may be attributable to the relatively short duration of runs (approximately 1–2 min), which demands maximal explosive exertion of power from the start of each jump and landing. Although these explanations remain speculative, they highlight potentially relevant mechanisms that could be further investigated in future research. The reason for the greater contribution of the W_Gly_ system and higher blood lactate concentration in SBX is because the nature of the discipline requires rapid acceleration from the start and repeated, continuous explosive movements (Zebrowska et al., 2012). In particular, instantaneous energy demand increases rapidly when passing obstacles at high speeds, resulting in focused use of the W_Gly_ system (Platzer et al., 2009). This enables a rapid supply of ATP but can also cause accumulation of lactate, leading to changes in the intramuscular environment and ultimately fatigue.

The structure of SS runs necessitated a longer run time than in SBX and short recovery intervals between skills (Federolf et al., 2008; Platzer et al., 2009). Plausibly, there are relatively low intensity gliding intervals or preparation intervals in between skill performance during the run, and the W_Oxi_ system is activated in these intervals to simultaneously enable energy supply and recovery. However, in our study, the difference in contributions of the W_Oxi_ system was not statistically significant. This could be because the total run times were short, at around 1–2 min, and the absolute contribution of the W_Oxi_ system remained consistently present in both disciplines (Beneke et al., 2004; Franchini, 2023). In particular, it is likely that both disciplines had similar patterns of energy utilization, with the W_PCr_ system acting as the main energy source during the first few seconds of the run, and glycolysis being activated after depletion of phosphocreatine (Gastin, 2001).

In addition, the snow during testing was mechanically compacted and had a high moisture content owing to above-zero temperatures, resulting in wet conditions. Such conditions can increase surface resistance and reduce glide efficiency, thereby requiring greater muscular force to maintain velocity. This, in turn, may elevate the metabolic cost of each run and increase reliance on glycolytic pathways (Wolfsperger, Meyer & Gilgien, 2021). Reviews of alpine skiing physiology also emphasize that complex environmental factors, including snow conditions, can substantially influence athletes’ physiological responses and performance outcomes.

Insofar as both disciplines are sports involved in complex activation of W_Oxi_ metabolism and anaerobic metabolism systems together, it is expected that the absolute contribution of the W_Oxi_ system would be greatest when the total run time is considered (Beneke et al., 2004; Franchini, 2023). This is consistent with previous studies showing that, in similar high-intensity intermittent sports like Olympic boxing, 60–90% of total energy is supplied by the W_Oxi_ system, and the focused use of anaerobic pathways is only observed at decisive moments (Beneke et al., 2004; Franchini, 2023). Our results also indicate that there was no significant difference between SS and SBX in terms of total W_Total_, suggesting that, despite differences in run times, the overall amount of energy consumed was similar (Wang, Zhong & Wang, 2023). SS had a longer run time than SBX, but included lower intensity intervals, and because the average intensity was higher in SBX, the W_Total_ was similar although the run time was shorter. In summary, both disciplines were largely dependent on W_Oxi_ metabolism as an energy source, and both involved strategic utilization of anaerobic systems for moment-to-moment explosive efforts (Platzer et al., 2009). However, the main finding of our study is that the relative contribution of these anaerobic systems is greater in SBX. This reflects that SBX is characterized by a higher average intensity throughout the run, which explains why the total energy expenditure was comparable to SS despite the shorter run duration.

In addition to conditioning and recovery strategies, discipline-specific competition strategies should also be considered. For example, in SBX, athletes must progress through multiple heats, making efficient recovery between rounds (*e.g.*, light cool-down, maintaining body warmth, and timely carbohydrate intake) particularly important. In contrast, SS relies on a single decisive performance, which emphasizes the importance of optimizing pre-competition routines and maintaining psychological regulation during the event.

This study has several limitations. First, the relatively small sample size of Tier 3 elite snowboarders may limit statistical power and the generalizability of our findings (McPhail et al., 2024). Second, because the measurements were conducted outdoors, environmental factors such as temperature and snow conditions may have influenced physiological responses despite careful calibration of the equipment (Buchheit & Laursen, 2013; Montoye, Vondrasek & Hancock, 2020; Poole & Jones, 2012; Seemann-Sinn et al., 2025). Third, although the VO_2_ Master Analyzer is validated for field use, short-duration high-intensity exercise may not fully capture absolute oxidative contributions. Finally, the simulated runs excluded rotational tricks and competitive race dynamics, which may not entirely reflect actual competition demands. Future studies should recruit larger and more diverse samples and apply ecologically valid competition settings to enhance generalizability (Merz et al., 2025).

## Conclusion

By comparing the energy system usage of the two snowboarding disciplines of SS and SBX, we observed clear differences in physiological demands between the disciplines. Although both disciplines depended on mixed energy systems, SBX demonstrated greater reliance on anaerobic glycolysis, highlighting the particular importance of this energy pathway in this discipline. In SS, while brief recovery intervals relied on the W_Oxi_ system, efficient W_Gly_ energy utilization was crucial for generating explosive power necessary for skill execution. Training that strengthens anaerobic glycolysis is essential for both disciplines. These findings could be used as basic data to help design more specific training strategies for each discipline. Future research should address the identified limitations and enhance generalizability through more precise and ecologically valid experimental designs.

##  Supplemental Information

10.7717/peerj.20948/supp-1 Supplemental Information 1 Raw data The measurements of oxygen uptake, heart rate, and blood lactate concentration from both snowboard slopestyle and snowboard cross trials. The data were used to estimate the energy system contributions and physiological profiles of the participants.

## Additional Information and Declarations

### Data Availability

The following information was supplied regarding data availability: The raw data is available in the Supplemental File.
